# Plant responses to limited aeration: Advances and future challenges

**DOI:** 10.1002/pld3.488

**Published:** 2023-03-26

**Authors:** Laura Dalle Carbonare, Juan de la Cruz Jiménez, Sophie Lichtenauer, Hans van Veen

**Affiliations:** ^1^ Department of Biology University of Oxford Oxford UK; ^2^ Graduate School of Bioagricultural Sciences Nagoya University Nagoya Japan; ^3^ Institute of Plant Biology and Biotechnology University of Münster Münster Germany; ^4^ Plant Stress Resilience, Institute of Environmental Biology Utrecht University Utrecht The Netherlands; ^5^ Groningen Institute for Evolutionary Life Sciences University of Groningen Groningen The Netherlands

**Keywords:** aerenchyma, ethylene, fermentation, flooding, hypoxia, mitochondria

## Abstract

Limited aeration that is caused by tissue geometry, diffusion barriers, high elevation, or a flooding event poses major challenges to plants and is often, but not exclusively, associated with low oxygen. These processes span a broad interest in the research community ranging from whole plant and crop responses, post‐harvest physiology, plant morphology and anatomy, fermentative metabolism, plant developmental processes, oxygen sensing by ERF‐VIIs, gene expression profiles, the gaseous hormone ethylene, and O_2_ dynamics at cellular resolution. The International Society for Plant Anaerobiosis (ISPA) gathers researchers from all over the world contributing to understand the causes, responses, and consequences of limited aeration in plants. During the 14th ISPA meeting, major research progress was related to the evolution of O_2_ sensing mechanisms and the intricate network that balances low O2 signaling. Here, the work moved beyond flooding stress and emphasized novel underexplored roles of low O2 and limited aeration in altitude adaptation, fruit development and storage, and the vegetative development of growth apices. Regarding tolerance towards flooding, the meeting stressed the relevance and regulation of developmental plasticity, aerenchyma, and barrier formation to improve internal aeration. Additional newly explored flood tolerance traits concerned resource balance, senescence, and the exploration of natural genetic variation for novel tolerance loci. In this report, we summarize and synthesize the major progress and future challenges for low O_2_ and aeration research presented at the conference.

## 1 INTRODUCTION

Oxygen and aeration limitation is a common aspect of plant life. It is found in dense, compact, and highly active tissues, but also at high elevation, atmospheric pressure can drop significantly. A well‐known cause of low aeration is flooding, which is common to many ecosystems. However, due to climate change, the frequency and intensity of rainfalls have and continue to increase dramatically (Hirabayashi et al., 2013), affecting ecosystems and imposing tremendous yield losses for flooding sensitive crops. These are troubling developments for our capacity to ensure food supply for an increasing world population. Unfortunately, most crops are not tolerant to floods. Furthermore, low oxygen tolerance is important for post‐harvest physiology.

During flooding, the pore spaces in soils are filled with water, and the plant shoots can become submerged. Gas diffusion in water is approximately 10,000 times slower than in air. Thus, flooding seriously impairs gas exchange between the plant and the environment. Additionally, oxygen (O_2_) in the soil is rapidly consumed by microorganisms and roots, which creates an anoxic environment. Low O_2_ availability during flooding seriously impairs plant function (Voesenek & Bailey‐Serres, 2015). In addition, the leaves of submerged plants face reduced availability of carbon dioxide (CO_2_), which leads to compromised photosynthesis and carbon starvation. This is often aggravated by murky water with low light penetration (Loreti et al. 2016). Bulky or compact tissues, such as fruits, seeds, and tubers, suffer similar O_2_ availability problems caused by diffusion limitations (van Dongen & Licausi, 2015). A lack of oxygen, the major electron acceptor at the mitochondria, drastically impairs redox balance and ATP generation. Hypoxic tissues will need to switch to internal organic electron acceptors, as implemented by ethanol and lactate fermentation to maintain redox balance and ATP regeneration. Additionally, O_2_ gradients and hypoxic niches in the plant play a key role in directing plant development, such as seedling establishment, meristem activity, and organ formation (Weits et al., 2021). Diffusion limitations also prevent the gaseous hormone ethylene to escape the plant, and therefore, ethylene is a powerful and reliable signal of aeration status that stimulates processes like adventitious rooting and aerenchyma formation (Loreti et al. 2016).

The International Society for Plant Anaerobiosis (ISPA) aims to understand the causes, responses, and consequences of limited aeration in plants and seeks to identify mechanisms through which plants acclimatize, adapt, or exploit an altered aeration status. At this year's society conference, held on 25–29 September 2022 at Kloster Banz, Germany, the recent research developments were shared. In this report, we highlight the key themes, progress, and challenges that were addressed. These include (1) the evolution of O_2_ sensing in and beyond the plant kingdom; (2) the intricate network that balances low O_2_ signaling; (3) the relevance of O_2_ in development of fruit, new organs, and seedling establishments; (4) developmental plasticity to improve flood tolerance by improving aeration or resource balance; and (5) the exploration of natural variation in low O_2_ responses within and beyond crops.

## 2 OXYGEN SENSING AND SIGNALING ACROSS KINGDOMS

**FIGURE 1:**
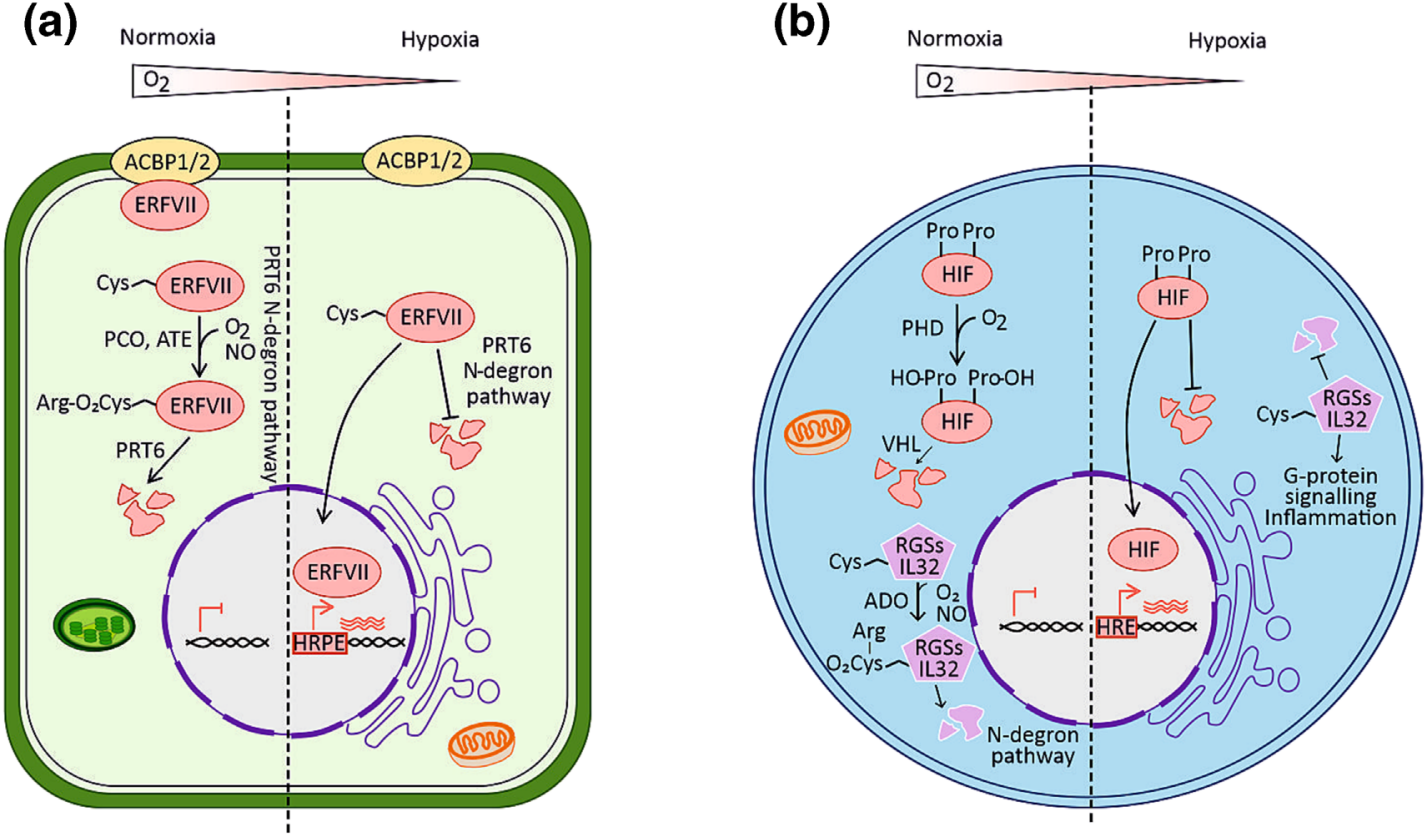
Comparison between the O_2_ sensing mechanisms in plants (angiosperm *Arabidopsis thaliana* ) and animals. (a) O_2_ levels in the plant cell are detected through the conditional degradation of constitutively expressed group VII of ethylene responsive factors (ERF‐VIIs) via the PCO branch of the PRT6 N‐degron pathway. In situations where O_2_ is available in the cell, after removing the initial Met, the Cys at the N‐terminus of ERF‐VIIs is oxidized to cys‐sulfinic acid by the PLANT CYSTEINE OXIDASE (PCO). Sequentially, the new N‐degron is arginylated by ARGININE TRANSFERASE (ATE) and ubiquitinated by ligase PROTEOLYSIS 6 (PRT6) to be target to the 26s proteasome for degradation. A fraction of the ERF‐VIIs remains sequestered at the plasma membrane bound to ACYL‐CoA BINDING PROTEINS 1 and 2 (ACBP1/2). Under hypoxic conditions, ERF‐VIIs are released from the plasma membrane and are no longer degraded due to the inhibition of PCO by the absence of O_2_. Stable ERF‐VIIs translocate to the nucleus where they bind a *cis*‐element (hypoxia responsive promoter element [HRPE]) on promoters of hypoxia inducible genes to initiate an acclimation response. (b) Similarly, O_2_ levels in animal cells are perceived through the HIF‐PHD‐VHL system. In aerobic conditions, the HYPOXIA INDUCIBLE FACTOR (HIF) is oxidized on two conserved Pro residues by PROLYL HYDROXYLASE DOMAIN (PHD) and targeted for proteasomal degradation by von Hippel–Lindau TUMOUR‐SUPPRESSOR PROTEIN (VHL) via the addition of ubiquitin units. On the contrary, under hypoxia, the oxygen sensor HIF is stable and translocated to the nucleus where it binds to specific hypoxia responsive promoters containing a hypoxia responsive element (HRE). In parallel, similarly to PCOs in plants, the enzyme CYSTEAMINE (2‐AMINOETHANETHIOL) DIOXYGENASE (ADO) is able to oxidize exposed N‐terminal Cys to cys‐sulfinic acid and target substrates (e.g., RGS4, RGS5, and IL32) for degradation depending on the O_2_ availability. Potential N‐terminal Cys‐degron transcription factor targets of ADO have not been identified so far.

In flowering plants, molecular O_2_ signaling occurs via the O_2_‐dependent stability of group VII of the ethylene responsive factor family (ERF‐VIIs), which are constitutively expressed transcription factors with a conserved N‐terminal motif starting with Met‐Cys (Nakano et al., 2006). This N‐terminus makes them a target of the Cys‐Arg branch of the N‐degron pathway in the presence of O_2_ and nitric oxide (NO). The ERF‐VII fraction that is free in the cytosol undergoes a series of enzymatic reactions where the initiating Met is removed through the activity of METHIONINE AMINOPEPTIDASE (MetAP) to reveal Cys (Gibbs et al., 2011; Licausi et al., 2011). The amino‐terminal Cys is then oxidized by PLANT CYSTEINE OXIDASEs (PCOs) and converted into cys‐sulfinic acid (Weits et al., 2014; White et al., 2017). This modified N‐terminal enables the subsequent Nt‐arginylation by ARGININE TRANSFERASE (ATE), which is then recognized by ligase PROTEOLYSIS 6 (PRT6) to ubiquitinate the ERF‐VIIs and targets them for proteasomal degradation (Graciet & Wellmer, 2010). Together with O_2_, NO is also required for degradation of Met‐Cys substrates in plants; however, it is yet unknown which component of the N‐degron pathway functionally depends on the presence of NO (Gibbs et al., 2014; Holdsworth et al., 2020). Simultaneously, ACYL‐CoA BINDING PROTEINS 1 and 2 (ACBP1/2) can sequester some ERF‐VIIs at the plasma membrane and protect them from degradation under aerobic conditions. During hypoxia, the lack of O_2_ prevents PCOs from oxidizing the N‐terminal cysteine, and therefore, proteolysis is averted. Additionally, the ERF‐VIIs from the plasma membrane are released. This allows the accumulation of stable ERF‐VIIs in the nucleus where they activate transcription of target genes, which include a set of conserved hypoxia responsive genes (HRGs) (Gasch et al., 2016; Kosmacz et al., 2015; Mustroph et al., 2009; Reynoso et al., 2019) (Figure 1a). PCO action is not specific to the N‐terminus of ERF‐VIIs but also mediates the O_2_‐dependent stability of other Cys‐terminal proteins. In plants, two additional targets of PCO have been identified, namely, VERNALISATION 2 (VRN2) (Gibbs et al., 2018) and LITTLE ZIPPER 2 (ZPR2) (Weits et al., 2019). An N‐terminal Met‐Cys does not guarantee targeting for degradation in the presence of O_2_ and NO. Sub1A‐1, an ERF‐VII in rice, is considered to remain stable because its N‐terminal sequence, containing the conserved Met‐Cys pattern, physically interacts with the Sub1A‐1 C‐terminus, which thereby would shield it from modifications (Lin et al., 2019).

The way in which species that are evolutionary distant from angiosperms sense O_2_ levels remains elusive but has gained the attention of the community in recent years, because species like bryophytes often thrive in highly humid and flood‐prone habitats, where O_2_ availability is limited. PCOs, the primary O_2_ sensors, appeared early during the colonization of land by plants; however, their transcriptional regulation by hypoxia was only acquired later by spermatophytes (Weits et al., 2022). Nonetheless, PCOs of bryophytes have been characterized as functional O_2_ sensors (Taylor‐Kearney et al., 2022). At the meeting, Monica Perri (University of Oxford, UK) presented work where she explored whether the bryophyte PCOs indeed exert a role during the acclimation to low O_2_. Regarding the ERF‐VII N‐terminal sequence, Laura Dalle Carbonare (University of Oxford, UK) suggested that angiosperms acquired their conserved feature (MCGGAI) in a stepwise manner along land plant evolution. Several studies show that *Arabidopsis* mutants for PCOs or plants overexpressing stable versions of ERF‐VIIs have drastic phenotypes with negative effects on the overall plant fitness and without improving plant tolerance to low O_2_ (Licausi et al., 2011; Masson et al., 2019). Nonetheless, Emily Flashman (University of Oxford, UK) presented how the availability of crystal structures for *Arabidopsis* PCO4 and PCO5 offers new ways to manipulate the O_2_ sensing mechanism by modifying the PCO affinity for O_2_, possibly without interfering with plant fitness (White et al., 2020).

The monitoring of internal O_2_ is also a well‐studied feature of animal cells. Both plant and animal responses to hypoxia are based on the O_2_‐dependent degradation of transcription factors, but the enzymatic pathways and the substrates are different. The O_2_ sensing in animal cells was firstly discovered in 2008 by Sir Peter Ratcliffe (University of Oxford, UK) and colleagues, and in 2019, it was awarded with the Nobel Prize in Physiology or Medicine (Kaelin et al., 2019). It is based on the O_2_ sensor PROLYL HYDROXYLASE DOMAIN (PHD), which in aerobic conditions catalyzes the hydroxylation of HYPOXIA INDUCIBLE FACTOR (HIF, two members HIF‐1a and HIF‐1β). The hydroxylation of conserved Pro residues of HIF is then recognized as a target for ubiquitination and proteasomal degradation (Kaelin & Ratcliffe, 2008) (Figure 1b). Under hypoxia, HIF stabilization induces transcriptional responses towards acclimation. The convergent evolution of the two mechanisms poses important questions regarding the advantage that such mechanisms provide to cells in response to low O_2_. In 2019, the animal counterpart of PCO was identified, namely, CYSTEAMINE (2‐AMINOETHANETHIOL) DIOXYGENASE (ADO), which has a similar enzymatic activity to that of PCO towards animal‐specific Met‐Cys initiating substrates (Masson et al., 2019). Why did animals prefer the HIF‐1a‐based system to sense O_2_ instead of the ADO‐based mechanism (homologous to the plant PCO/PRT6 O_2_ sensing mechanism)? Vinay Shukla (University of Oxford, UK) approached this question by swapping the sensors between the two organisms with the future goal to design synthetic systems to regulate responses to low O_2_ to grasp essential properties of O_2_ sensing mechanisms. In conclusion, as Sir Peter Ratcliffe emphasized at the meeting, all eukaryotic kingdoms use protein oxidation as a signaling mechanism; however, still very little is known about the inter‐relationship between them. Moreover, evolutionary distant organisms could provide a source of yet undiscovered alternative oxygen sensors.

## 3 DOSAGE CONTROL IN PLANT HYPOXIA SIGNALING

**FIGURE 2:**
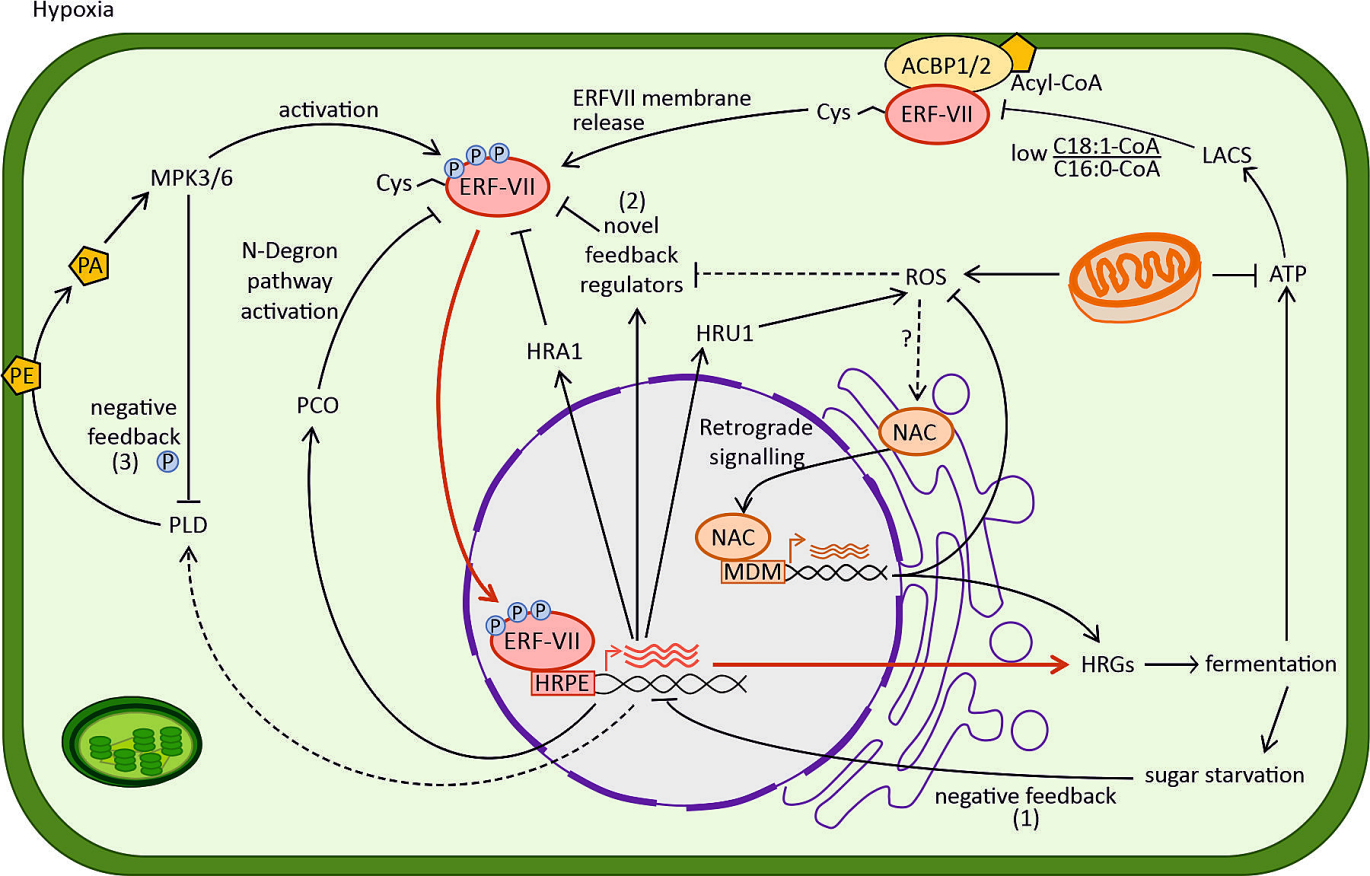
Dosage control of ERF‐VII signaling during hypoxia acclimation. During hypoxia, ERF‐VIIs are no longer degraded via the PRT6 N‐degron pathway; in parallel, low levels of ATP (as consequence of mitochondrial dysfunction) reduce LONG‐CHAIN ACYL‐CoA SYNTHETASE (LACS) activity and alter the fatty acid composition of the plasma membrane (higher ratio of C18:1‐CoA over C16:0‐CoA), inducing the release of ERF‐VIIs from ACYL‐CoA BINDING PROTEINS 1 and 2 (ACBP1/2). These two events result in ERF‐VII accumulation and translocation to the nucleus where HYPOXIA RESPONSIVE GENES (HRGs) are activated by ERF‐VIIs binding to the hypoxia responsive promoter element (HRPE) motif in the promoter regions (as shown by the red arrows). Among the set of HRGs, those encoding for proteins involved in fermentation are essential to provide an alternative source of ATP to the O_2_‐dependent production by mitochondrial electron transport chain (mETC). However, high fermentation rate leads to higher carbon consumption and eventually sugar starvation, whose dramatic effects are prevented by negative feedback regulation (1) on the same fermentation‐related HRGs. In addition, accumulation of stable ERF‐VIIs is prevented over time by the hypoxia‐induced negative regulator HRA1 and PCO (which reactivates the N‐degron pathway and ERF‐VII degradation upon reoxygenation) and novel feedback loops (2). Nonetheless, the hypoxia‐induced PHOSPHOLIPASE D (PLD, dashed arrow), through the conversion of phosphatidylethanolamine (PE) into phosphatidic acid (PA), activates MITOGEN‐ACTIVATED PROTEIN KINASEs (MPK3/6), which leads to ERF‐VII phosphorylation and ultimately enhances hypoxia signaling. At the same time, it inactivates PLD itself via phosphorylation (3). Low O_2_ conditions increase generation of reactive oxygen species (ROS), via the accumulation of HYPOXIA‐RESPONSIVE UNIVERSAL STRESS PROTEIN 1 (HRU1) and mitochondrial malfunctioning. ROS accumulation triggers retrograde signaling via the cleavage of NAC transcription factors from the ER membrane, enabling translocation to the nucleus, where it binds to a MITOCHONDRIAL DYSFUNCTION MOTIF (MDM) in promoters of target genes. In addition, high levels of ROS inhibit novel feedback regulators (2) with effect on the stability of ERF‐VIIs.

The hypoxia responsive genes (HRGs) regulated by ERF‐VIIs under hypoxia include those required for fermentative metabolism essential for energy supply and redox management and not surprisingly have received much research attention (Mustroph et al., 2009). Activation of HRGs via ERF‐VIIs seems straightforward. However, negative feedback regulation by the ERF‐VII target HYPOXIA RESPONSIVE ATTENUATOR 1 (HRA1) was previously shown (Giuntoli et al., 2014), and strong interaction with ROS signaling networks was established by another ERF‐VII target HYPOXIA‐RESPONSIVE UNIVERSAL STRESS PROTEIN 1 (HRU1) (Gonzali et al., 2015). The work presented at the meeting covered many novel discoveries and the expansion of our knowledge around an intricate network of ERF‐VII regulators that could allow for precise dosage control to prevent excessive hypoxic gene induction and match the hypoxic response to the physiological status of the cell (Figure 2).

A first example of this dosage control of ERF‐VII activity in *Arabidopsis* was unraveled by Romy Schmidt‐Schippers (Bielefeld University, DE) and coworkers. They showed how the ERF‐VII mediated hypoxia induction of *SIMILAR TO RCD ONE 5* (*SRO5*) during prolonged hypoxia leads to an inhibition of ERF‐VII mediated transcriptional induction of target genes. Additionally, they showed that RADICAL‐INDUCED CELL DEATH 1 (RCD1), which increases under hypoxia, represses ERF‐VIIs and other transcription factors already early in hypoxia but is degraded in a redox‐dependent manner when exposed to ROS, a key signature of prolonged hypoxia (Pucciariello & Perata, 2017). Regarding monocots, Ming‐Che Shih (Academia Sinica, TW) showed how the O_2_ labile ERF‐VIIs in *Brachypodium distachyon* activate O_2_ stable ERFs that in turn suppress ERF‐VIIs to prevent a runaway hypoxic response. This contrasts to the behavior of SUB1A in rice. Here, SUB1A, an O_2_ stable ERF‐VII, stimulates the expression of O_2_ labile ERF‐VIIs to enhance O_2_ sensitivity (Lin et al., 2019).

ERF‐VIIs are key to initiate fermentation to enable generation of ATP during hypoxia. As fermentative metabolism has a very low ATP yield, it requires high carbon consumption rates to be effective and hence is limited by sugar availability. Indeed, sugar starvation dampens the transcriptional activation of HRGs by ERF‐VIIs (Loreti et al., 2018). Alicja Kunkowska (Sant'Anna School of Advanced Studies, IT) presented evidence that a key ERF‐VII in *Arabidopsis* is phosphorylated in an energy signaling‐dependent manner to fine‐tune hypoxic transcriptional signaling (Kunkowska et al., 2023). In contrast, a lack of ATP has also been proposed as a driver of ERF‐VII activation by affecting acyl‐CoA metabolism such that it triggers ERF‐VII translocation to the nucleus (Schmidt et al., 2018; Zhou et al., 2020). Similarly, Shi Xiao (Sun Yat‐sen University, CN) showed that under hypoxia, phosphatidic acid (PA) accumulation negatively affects hypoxic signaling by compromising membrane integrity, but at the same time, it triggers a phosphorylation cascade via MITOGEN‐ACTIVATED PROTEIN KINASEs (MPK3/6) to RAP2.12, which results in enhanced hypoxic response (Zhou et al., 2022). This seeming contrast in relationship between energy starvation and activation/inhibition of ERF‐VII activity could potentially be explained by different requirements in early and long‐term hypoxia (Cho et al., 2021).

Impaired mitochondrial function and associated retrograde signaling result in a strong overlapping transcriptional response compared to O_2_ deprivation (Meng et al., 2020; Wagner et al., 2018, 2019). The retrograde ANAC transcription factor NAC017, which moves from the ER to the nucleus during mitochondrial dysfunction (Ng et al., 2013) was identified as important for flooding acclimation (Bui et al., 2020; Meng et al., 2020). Similarly, Tilo Renziehausen and coworkers (Bielefeld University, DE) showed that under hypoxia, ANAC013 another retrograde signaling transcription factor (De Clercq et al., 2013), is released from the ER to nucleus where it regulates key HRGs whose promoters contain a specific mitochondrial dysfunction motif (MDM). It is yet unclear whether retrograde signaling acts as a parallel pathway to the classical N‐degron‐mediated O_2_ signaling or whether both pathways are required to achieve an optimal hypoxic response that not only considers O_2_ availability but also mitochondrial functioning.

The multitude of feedback mechanisms and integration between pathways that is emerging indicates that it is very important to prevent excessive ERF‐VII signaling and keep it in tune with the physiological status of the cell and its organelles. The above examples merely scratch the surface of potential interactors of metabolism and low O_2_ signaling mechanisms. For instance, Sergey Shabala (University of Tasmania, AU) highlighted ion channels that could act as O_2_ sensors and the relevance of γ‐aminobutyric acid (GABA) as metabolic signature of hypoxia that affects K^+^ homeostasis and the regulation of membrane potential and central ROS/Ca^2+^ signaling hubs (Wang et al., 2017; Wu et al., 2021). Hypoxia also sees dynamics in NO levels through the reduction of nitrite at the electron transport chain and its recycling via phytoglobin that improves hypoxia tolerance and respiratory flux (Gupta et al., 2005, 2020; Mugnai et al., 2012). NO is further required for N‐degron‐based degradation of ERF‐VIIs (Gibbs et al., 2014) and interconnected with ethylene signaling (Hartman et al., 2019; Manac'h‐Little et al., 2005). At this ISPA conference, Vajiheh Safavi‐Rizi (Leipzig University, DE) presented a potential role of AMIDOXIME REDUCING COMPONENT (ARC) as a new player of NO metabolism in *Arabidopsis* (Chamizo‐Ampudia et al., 2016).

The high integration and multitude of signals potentially obscure the dynamics of individual components of the N‐degron pathway in initiating low O_2_ responses. Beatrice Giuntoli (University of Pisa, IT) and coworkers were able to circumvent such limitations by introducing the core O_2_ signaling components of plants into yeast (Puerta et al., 2019). This allowed them to test the role of individual players and led to the finding that HRG induction driven solely by ERF‐VII nuclear accumulation from de novo synthesis responds to an O_2_ decline in already 5 min. In *Arabidopsis*, Markus Schwarzländer and Sophie Lichtenauer (University of Münster, DE) gained new insights into ATP levels, NAD redox dynamics, and oxidative stress during acclimation to varying concentrations of O_2_ by using fluorescent biosensors with high time resolution, which will further aid in characterizing the dynamics of low O_2_ signaling (Wagner et al., 2019). However, we have identified many coregulators and feedback mechanisms acting on ERF‐VIIs (Giuntoli & Perata, 2018), with recuring themes being feedback regulation, integration with resource and energy systems, and retrograde signaling. Dosage and temporal dynamics of hypoxia are clearly important, along with established cell type variation in hypoxic responses (Mustroph et al., 2009). The future challenge will be to integrate, add weight and conditionality to each of these processes to build a robust model of O_2_ signaling *in planta*.

## 4 THE ROLE OF LOW OXYGEN SIGNALING BEYOND FLOODING

As described earlier, a key part of HRGs is the induction of the ethanol fermentation machinery, and a reduced or stronger HRG signature leads to corresponding tolerance to a hypoxic treatment (Gibbs et al., 2011; Hartman et al., 2019; Hinz et al., 2010; van Veen et al., 2013). However, under flooding conditions, a relationship between ERF‐VIIs and tolerance is either negative, positive, or absent (Giuntoli et al., 2014; Licausi et al., 2011; Riber et al., 2015; Sasidharan et al., 2013; Tang et al., 2021; van Veen et al., 2016). Three explanations for the apparent disconnect between the manipulation of ERF‐VII signaling and flood tolerance are that (1) low O_2_ is mostly restricted to the underground parts of the plant (Vashisht et al., 2011), that (2) capacity for fermentation is dominated by sugar supply rather than fermentative gene induction (Magneschi et al., 2009; Santaniello et al., 2014), and/or that (3) major transcriptomic changes during flooding are strongly related to darkness, starvation, and mitochondrial retrograde signaling, indicating that the majority of acclimation during flooding occurs outside ERF‐VIIs (Meng et al., 2020; van Veen et al., 2016). Indeed, other roles beyond flooding stress are now becoming the focus of low O_2_ signaling.

O_2_ levels are not distributed uniformly within the plant, also when grown under favorable conditions. Hypoxic niches and strong gradients form particularly in meristems, compact seeds, and bulky fruits and have an important role for the development of the whole plant (Shukla et al., 2019; van Dongen & Licausi, 2015; Weits et al., 2019). The exact cause of *in planta* O_2_ gradients is not always straightforward; key aspects relate to the cellular arrangement, diffusion barriers, and O_2_ production or consumption rates. Regarding tomato fruit, Hui Xiao (University of Leuven, BE) presented a model based on a pore network, computed by X‐ray micro‐computed tomography (μ‐CT) (Xiao et al., 2021) and tissue‐specific respiration rates that explains the lower O_2_ levels in the gel/seed part and the higher O_2_ levels in the pericarp supplied via the pedicel scar. Similarly, Hardy Rolletschek (Leibniz Institute of Plant Genetics and Crop Plant Research, DE) showed how a void space along the pericarp contributes to O_2_ supply in maize kernels, which could aid in O_2_ delivery to affect kernel physiology.

Reducing O_2_ is an established method to delay fruit ripening and prolong storage, but at the risk of damaging the fruits. In hypoxic‐stored apples, ERF‐VIIs are stabilized and activated (Cukrov et al., 2016), similarly to *Arabidopsis* ERF‐VIIs, as shown by Suzanne Pols (KU Leuven, BE). The gaseous hormone ethylene is another key driver of fruit ripening. Ethylene diffusion follows similar physical rules as O_2_, but because it is produced rather than consumed by living tissues, it tends to accumulate in poorly aerated tissues. Benedetto Ruperti (University of Padua, IT) presented how ethylene production in fruits depends on O_2_ availability and showed how during apple storage a complex interaction of O_2_ and ethylene‐dependent and independent effects govern apple metabolism.

Meristems, both in the root and the shoot, also are hypoxic niches. However, in contrast to fruits and seeds, it is not clearly defined how the root and apical meristems generate and preserve hypoxia. Viktoriia Voloboeva (Utrecht University, NL) presented possible mechanisms to maintain a hypoxic niche, with emphasis on a role that cuticles of the outer cell layer might play. In the shoot apex, local hypoxic conditions allow stabilization of the locally expressed N‐degron target ZPR2, which helps to maintain meristem activity and define leaf development (Weits et al., 2019). By genetically inhibiting O_2_‐mediated degradation of N‐degron proteins, Daan Weits and Gabrielle Panicucci (Utrecht University, NL) showed that leaf development displayed morphological phenotypes of delayed differentiation or possibly even de‐differentiation. Where low O_2_ delays fruit development (Boeckx et al., 2019), leaf formation and lateral root emergence are stimulated and require low O_2_ (Shukla et al., 2019; Weits et al., 2019).

Besides development, imposing low O_2_ signaling, either externally or genetically, prevents photomorphogenesis of an emerging seedling. Because chlorophyll homeostasis during germination in the dark is O_2_ dependent, O_2_ received from the air or from early photosynthesis is a key requirement to kick‐start seedling establishment (Abbas et al., 2015). This phenomenon can cause problems for plants grown at high altitudes where O_2_ partial pressures are lower. To compensate for low partial O_2_ pressure, high‐altitude *Arabidopsis* ecotypes display a more sensitive response to O_2_ to adjust signaling during germination in the dark correspondingly, as described by Michael Holdsworth (University of Nottingham, UK) (Abbas et al., 2022).

The relevance of hypoxia not only as a metabolic stressor but also as a signaling molecule (Weits et al., 2021) highlights the need to understand the dynamics and spatial patterns of O_2_ availability, but measurement of O_2_ concentrations with spatiotemporal resolution within tissues remains challenging. Regarding O_2_, microelectrodes, modeling approaches and HRG expression provide our current knowledge base. The ISPA conference highlighted how the community is developing the use of sensor foils, genetically encoded O_2_‐sensitive fluorophores or fluorogenic probes to noninvasively characterize O_2_ gradients within the plant with high spatial resolution and in pursuit of temporal sensitivity (Rolletschek & Liebsch, 2017; Weits,  2021). Similar to O_2_, the gaseous hormone ethylene, despite different production/consumption and sensing mechanisms, is affected by the same physicochemical constraints in aeration imposed by flooding or tissue geometry. Hence, for full understanding of aeration‐dependent metabolism and development, establishing toolsets such as those developed for O_2_ will be crucial for ethylene too.

## 5 TOWARD FLOOD TOLERANCE: IMPROVED INTERNAL AERATION

**FIGURE 3:**
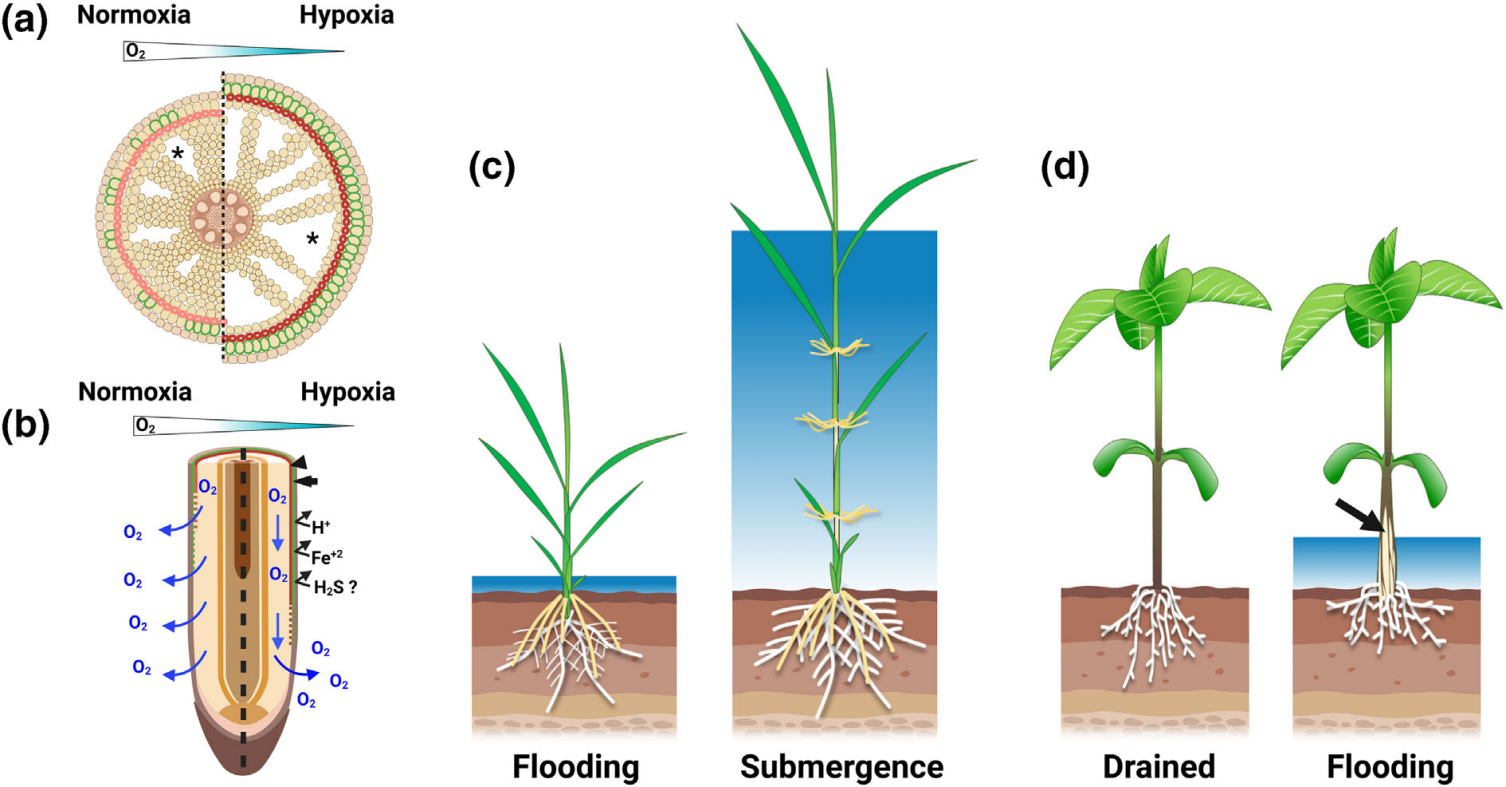
Key anatomical and morphological changes in response to flooding and partial submergence. When the soil is flooded, (a) rice develops new adventitious roots with larger diameters and increased aerenchyma spaces. Moreover, the cell walls of the exodermal and sclerenchyma layers are heavily impregnated with suberin and lignin, respectively. Black arrows and arrowheads in (b) and (c) indicate suberized exodermal cells (green color) and lignified sclerenchyma (red color), respectively. Asterisks indicate aerenchyma spaces. The increased deposition of these polymers in cell walls enable (b) the formation of apoplastic barriers impeding radial oxygen loss and restricting the entry of toxic compounds (i.e., Fe2+, organic acids) produced in flooded soils. During partial submergence, (c) deepwater rice elongates the internodes in order for the leaves to reach air. Similarly, in flooded or partially submerged soils, (d) legumes develop hypertrophied lenticels with extensive secondary aerenchymatous phellem above the water surface to enable O2 diffusion down to submerged tissues. Black arrow in (d) points to secondary aerenchyma formation. Created with BioRender.com.

During flooding, shortage of O_2_ in the belowground parts of the plant limits root respiration, growth, and nutrient uptake. Flood‐adapted plants such as rice circumvent O_2_ shortage through effective internal aeration during prolonged flooding conditions and so guarantee plant survival. A key trait for improved internal aeration is the combination of radial expansion of the root cortex and aerenchyma formation (Figure 3a; Yamauchi, Abe, et al., 2019). Constitutive and inducible aerenchyma formation in rice roots is mediated by auxin (Yamauchi et al., 2019) and ethylene (Drew et al., 1979), respectively. The molecular mechanisms that underlie root cortex expansion in response to flooding remain unclear but are under investigation by Takaki Yamauchi (Nagoya University, JP). He presented a rice mutant deficient in auxin signaling, which compared with the wild type, has smaller cortex area and reduced cortex expansion in response to flooding and can serve as a model to understand the genetic regulation of the cortex expansion during flooding.

In addition to increased root diameter and aerenchyma, some flood tolerant plants (e.g., rice) form apoplastic barriers in roots that impede radial O_2_ loss (ROL) to the anoxic rhizosphere during soil flooding. The barriers enhance longitudinal O_2_ transport along the length of the root toward the tip (Figure 3b). The formation of these barriers is associated with increased impregnation of suberin and lignin polymers in cell walls of the outer part of the root (i.e., epidermis, exodermis, and/or sclerenchyma; Abiko et al., 2012; Jiménez et al., 2019; Kotula et al., 2009). These polymers act as physical barriers impeding the apoplastic movement of gases and solutes from roots to soils and vice versa. At the meeting, several speakers (Ole Pedersen and Lucas Peralta, University of Copenhagen, DK; Juan Jiménez, Nagoya University, JP) emphasized the relevance of this trait. Recent progress and the future challenges concern the identification of the genetics and chemical composition of these barriers and the importance of this trait in multiple stress conditions (e.g., flooding, drought, and toxic soils).

The barriers to ROL also impede the entry of toxic compounds produced in flooded soils (i.e., organic acids, Colmer et al., 2019; Fe^+2^, Jiménez et al., 2021; H^+^, Peralta Ogorek et al., 2021). Moreover, the barriers to ROL also restrict radial water loss and thereby slow down tissue desiccation (Peralta Ogorek et al., 2021; Song et al., 2022), which extend the importance of this trait for developing crops adapted to drought conditions. In line with this, Julia Bailey‐Serres (UC Riverside, US) in her closing talk indicated that exodermal cells from roots of rice grown in water deficit conditions exhibited a pronounced upregulated pattern for suberin biosynthesis genes. MYB and NAC transcription factors are key candidates in modulating the gene regulatory network of suberin formation in rice (Reynoso et al., 2022). This multifaceted root characteristic (i.e., barriers to ROL with increased suberization) appears as a particularly promising trait for breeding purposes because during normal cultivation cycles plants can experience temporal periods of both soil flooding and droughts.

In lowland regions or during periods of heavy rainfalls, water levels can rise to cause partial or even complete plant submergence. Deepwater rice varieties adapt to submergence by elongating the internode to keep part of the shoot above the water surface (Figure 3c; Catling, 1992; Hattori et al., 2009). This allows snorkeling behavior where diffusion of gases through the internode cavity and aerenchyma sustain aeration of the submerged parts of the plant. As shown by Keisuke Nagai (Nagoya University, JP), wetland monocots possess significantly higher porous spaces in their nodes compared with dryland monocot species, which further facilitates O_2_ diffusion when partially submerged. Risa Tanaka, Keisuke Nagai, and Motoyuki Ashikari (Nagoya University, JP) presented their progress on the physiological and molecular mechanisms of internode elongation in deepwater rice. Internode elongation and plant length under submergence track the increases in the water level to keep the shoot apex aerated (see supplementary movie 1 in Hattori et al., 2009), and this elongation is usually fast during the first days of complete submergence but slows down thereafter (Catling, 1992). It is still unknown whether the internode elongation during submergence is indeterminate. Risa Tanaka (Nagoya University, JP) indicated that a new internode (young) develops and elongates only after the previous (older) internode has stopped elongating. Moreover, differences in O_2_ partial pressures between younger and the following older internode suggest that O_2_ gradients could also play a role in internode elongation in addition to other key regulators such as ethylene and gibberellins (Hattori et al., 2009; Nagai et al., 2020). Such differences in O_2_ diffusion and concentration between internodes can be influenced by the anatomical characteristics of nodes.

Soybean displays a different strategy to improve aeration when challenged by soil flooding and/or partial submergence by forming hypertrophied lenticels allowing high O_2_ diffusion into the stem and increased secondary aerenchymatous phellem to facilitate O_2_ diffusion toward the submerged parts of the plant (Figure 3d; Shimamura et al., 2010; Takahashi et al., 2018). Using soybean as a model, Hirokazu Takahashi (Nagoya University, JP) identified specific genes associated with key metabolites of the triterpenoids required in secondary aerenchymatous phellem to improve internal aeration (Takahashi et al., 2022).

Improved aeration and limited ROL are key traits for flood tolerance; however, the formation of these structures is developmentally complex and tissue and age specific and requires cell type specific responses. The leveraging of cell type resolution, either through laser capture microdissection (Nakazono et al., 2003) or genetically encoded technologies like INTACT (Isolation of Nuclei Tagged in Specific Cell Types) and TRAP (Translating Ribosome Affinity Purification) (Reynoso et al., 2022), has and will continue to be key to systematic progress in our understanding of the plasticity in aeration traits in our major crops such as rice and soybean.

## 6 TOWARD FLOOD TOLERANCE: AGE‐SPECIFIC SENESCENCE

Anoxic and hypoxic treatments tend to rapidly stress and kill flooding‐sensitive plants, and any adaptive mechanisms to this treatment delay, rather than prevent death. Flooding, either waterlogging or partial or complete submergence, when no improved internal aeration can be achieved (see section above), typically leads to stunted performance or a more gradual demise of the plant. In such conditions, the senescence of the leaves is often used as a representative trait for tolerance. Also, the stressful recovery period following de‐submergence can trigger senescence of the leaves (Fukao et al., 2012; Yeung et al., 2018).

Senescence is a natural process toward the end of leaf's lifetime, where controlled breakdown of the leaf allows reuse of valuable nutrients either in new leaf growth or seed filling. Initiation and advancement through senescence is highly controlled and in addition to old age is also induced by darkness, too high or low sugar availability, and abiotic and biotic stressors (Rankenberg et al., 2021). Tom Rankenberg (Utrecht University, NL) and colleagues showed that under dark submergence, senescence progresses sequentially from old to young leaves and that the youngest primordia and shoot meristem survive flooding the longest. Using *Arabidopsis* as a model, they showed that ethylene, despite accumulating throughout the plant during flooding, is key to the sequential nature of senescence by age‐dependently phosphorylating, and thereby activating, the positive regulator of senescence ORESARA1 (Rankenberg et al., 2022). This regulation of submergence‐induced age‐dependent senescence contrasts from age effects under favorable conditions that are dominantly driven by microRNA regulation of *ORE1* (Li et al., 2013). In rice, SUB1A not only suppresses elongation to improve tolerance (Fukao et al., 2006; Fukao & Bailey‐Serres, 2008), it also limits leaf senescence (Alpuerto et al., 2016; Fukao et al., 2012). As Takeshi Fukao (Fukui Prefectural University, JP) presented at the conference, also SUB1A is induced by ethylene (Fukao et al., 2006) and also predominantly acts in the youngest leaves (Alpuerto et al., 2022).

Plants that inhibit senescence and stay greener during and after flooding, such as SUB1A rice, *oresara1*, ethylene signaling mutants, and tolerant *Arabidopsis* accessions, significantly perform better following flooding stress (Fukao et al., 2012; Rankenberg et al., 2022; Yeung et al., 2018). However, senescence is essential to remobilize valuable resources, and lack of senescence might compromise the long‐term tolerance of the meristem and undeveloped leaves that are natural sink tissues and might rely on resource remobilization. Hans van Veen (Utrecht University, NL) presented stable isotope labelling experiments demonstrating that indeed the old senescing leaves supply the meristem and undeveloped leaves with especially carbon rich amino acids. However, this did not contribute to long‐term tolerance of the plant, possibly because of the capacity of these sink tissues to engage into strong quiescence. The absence of a relationship between long‐term tolerance and senescence provides us opportunities to use inhibition of senescence as a trait to improve short to medium‐term flood tolerance without severe trade‐offs. Senescence is a highly complex and controlled process that seems independent of low O_2_ signaling, but rather underlies a network of ethylene, ABA, cytokinin, and ROS and intersects with age. Understanding and manipulating this network will be a key aspect of moving toward flood‐tolerant crops.

## 7 TOWARD FLOOD TOLERANCE: FORWARD GENETICS AND WILD SPECIES TO IDENTIFY TOLERANCE TRAITS

There is a wealth of genetic variation within rice germplasms (Ashikari & Matsuoka, 2006) that has been effectively exploited with forward genetics to identify key loci for flood tolerance. Indeed, SUB1A proved highly effective in tolerance to flash flooding (Fukao et al., 2006; Xu et al., 2006), and *OsTPP7* and *OsGF14h* improve tolerance and capacity of anaerobic germination (Kretzschmar et al., 2015; Sun et al., 2022). Apart from rice, true flood tolerance traits are not abundant among our major crops, hampering the identification of strong quantitative trait loci (QTLs). Nonetheless, some variation in flood tolerance exists, which does provide opportunities to identify existing genetic components that aid performance when confronted with floods.

In maize, the QTL *Subtol6* was able to explain 22% of variation in flood tolerance variation (Campbell et al., 2015). Two key candidate genes underlying this QTL are thought to improve tolerance by enhanced *PHYTOGLOBIN* transcription and key regulators that reduce senescence. At the meeting, Henry Nguyen (University of Missouri, US) shared extensive work on soybean genetics where they discovered a QTL that leads to enhanced root growth both in length and the number of root tips independent of waterlogging treatment. Fine‐mapping identified key auxin signaling players to mediate this tolerance trait (Ye et al., 2018). Another forward genetic approach toward crop‐tolerant traits and genes was presented by Chiara Pucciariello (Sant'Anna School of Advanced Studies, IT), where she explored the capacity of barley to germinate after a flooding event and found novel marker–trait associations (MTAs) through genome wide association mapping.

Ultimately, these forward genetic screens are limited by the poor tolerance extremes found within the crop populations. This was counteracted by introgression lines between maize and the flood tolerant wild relative *Zea* nicaraguensis, which helped to find and introduce key mediators of aerenchyma formation and ROL barriers into maize (Gong et al., 2019; Watanabe et al., 2017). Also, the many wild relatives of rice that are highly flood tolerant, often independent of SUB1A, represent a powerful resource to find novel tolerance regulators (Niroula et al., 2012). With the development of sequencing technology, our palette of species has increased, and we have been able to characterize wild species not only at a physiological level but also at a molecular genetic level (Kim et al., 2018; Müller et al., 2021; Nakayama et al., 2014; van Veen et al., 2013). At the meeting, we saw such efforts taken beyond transcriptomic assessment of a single or pair of species. Angelika Mustroph (University Bayreuth, DE) showed how sampling a multitude of species from a tolerant sublineage of the Brassicaceae identified key molecular players that could be helpful for underwater photosynthesis. Dana MacGregor (Rothamsted Research, UK) focused instead on the troublesome weed black grass that performs well in waterlogged conditions. By creating novel genetic resources of varying populations, she aims to provide the tools to decipher how waterlogging tolerance is acquired. Angelina Jordine (Aachen University, DE) took her study object to the extreme by dissecting the behavior of the flooding and salt‐tolerant *Salicornia europaea*. These undomesticated plants can provide us with essential understanding on how natural selection enforced flood tolerance and therefore can lead us to the key and feasible genetic changes required to create flood tolerance in crops.

## 8 FUTURE PERSPECTIVES

In a common goal to understand the regulation and relevance of aeration status in plants, the conference united a wide variety of expertise ranging from the cell to organism level. Moreover, we were blessed with numerous online contributions and active worldwide participation. The accessibility and broad expertise of the conference stimulated lively discussion and an integrative treatise of the topics.

The presented work expanded our knowledge of the intricate regulatory network surrounding low O_2_ signaling in plants, in part by leveraging alternate model systems such as yeast and exploring an evolutionary wide range of species. Low O_2_ signaling is not only relevant for metabolic acclimation but also plays a key role in development. The distribution of O_2_ levels across tissues is dependent on tissue geometry, cellular respiration, and diffusion barriers. Therefore, O_2_ levels vary across plant development but also play an important role in organizing tissue development. To fully understand these processes, it is essential to obtain a cellular resolution of O_2_ levels *in planta* and get a good grasp on the dose‐dependent effects of O_2_ on downstream signaling and plant performance. A future challenge is to include ethylene and other underexplored gaseous components such as CO_2_ as potential signals for aeration status in this equation, because their gaseous nature means it also provides a signal for aeration status. As such, ethylene already proved to be key driver of adaptive traits such as aerenchyma formation, adventitious root formation, and underwater shoot elongation (Jackson, 2008; Lin et al., 2023; Lorbiecke & Sauter, 1999; Sauter, 2013). Much work was shown on the integration and processing of the primary signals, but here many questions remain, especially regarding players such as NO, GABA, and K^+^ homeostasis.

The meeting featured a lot of work on *Arabidopsis* (43% of all talks and posters presented), of which a considerable amount was in vitro based experiments. Where controlled growth conditions provide experimental benefits, future challenges will be to move more toward understanding of flooding acclimation mechanisms and potential traits to improve tolerance in crops other than rice (only 17% of all talks and posters presented) on the one hand and research on field conditions on the other. There is also a need to study the interactions of flooding and other stresses such as drought, salinity, nutrient availability, or heavy metals that could occur simultaneously or sequentially in the field, as well as the contribution of soil and microbiome to the overall plant tolerance to flooding.

In contrast to *Arabidopsis*, the rice germplasms display a full suite of adaptive traits, many associated with improving aeration status. Here, the formation and performance of ROL barriers, not present in *Arabidopsis*, was a major topic at the conference. ROL barriers not only aid flood tolerance but also resistance to other stresses. Future challenges will be to translate tools and knowledge developed in *Arabidopsis* and rice toward flooding‐sensitive staple crops. At the ISPA22 conference, we heard about specific examples of aeration traits developed in response to flooded conditions, which improved tolerance in soybean. Nonetheless, universal traits such as senescence regulation and growth cessation could prove a more suitable avenue for implementing flood tolerance into crops that are unamendable to aeration improving traits. To follow the natural path toward flood tolerance, we should turn to wild species from wet habitats. The amphibious lifestyle evolved many times (Cook, 1999), and the work on these non‐model species will help us to decipher the independent routes to flood adaptation and could further aid us toward flood‐tolerant crops.

## AUTHOR CONTRIBUTIONS

All authors contributed equally and are listed alphabetically.

## CONFLICT OF INTEREST STATEMENT

The Authors did not report any conflict of interest.
